# A case for revisiting peer review: Implications for professional self-regulation and quality improvement

**DOI:** 10.1371/journal.pone.0199961

**Published:** 2018-06-28

**Authors:** Terry E. Hill, Peter F. Martelli, Julie H. Kuo

**Affiliations:** 1 Hill Physicians Medical Group, San Ramon, California, United States of America; 2 Center for Catastrophic Risk Management, University of California, Berkeley, California, United States of America; 3 Sawyer Business School, Suffolk University, Boston, Massachusetts, United States of America; Harper University Hospital, UNITED STATES

## Abstract

**Background:**

Quality improvement in healthcare has often been promoted as different from and more valuable than peer review and other professional self-regulation processes. In spite of attempts to harmonize these two approaches, the perception of dichotomous opposition has persisted. A sequence of events in the troubled California prison system fortuitously isolated workforce interventions from more typical quality improvement interventions. Our objectives were to (1) evaluate the relative contributions of professional accountability and quality improvement interventions to an observed decrease in population mortality and (2) explore the organizational dynamics that potentiated positive outcomes.

**Methods:**

Our retrospective mixed-methods case study correlated time-series analysis of mortality with the timing of reform interventions. Quantitative and qualitative evidence was drawn from court documents, public use files, internal databases, and other archival documents.

**Results:**

Change point analysis reveals with 98% confidence that a significant improvement in age-adjusted natural mortality occurred in 2007, decreasing from 138.7 per 100,000 in the 1998–2006 period to 106.4 in the 2007–2009 period. The improvement in mortality occurred after implementation of accountability processes, prior to implementation of quality improvement interventions. Archival evidence supports the positive impact of physician competency assessments, robust peer review, and replacement of problem physicians.

**Conclusions:**

Our analysis suggests that workforce accountability provides a critical quality safeguard, and its neglect in scholarship and practice is unjustified. As with quality improvement, effective professional self-regulation requires systemic implementation of enabling policies, processes, and staff resources. The study adds to evidence that the distribution of physician performance contains a heterogeneous left skew of dyscompetence that is associated with significant harm and suggests that professional self-regulation processes such as peer review can reduce that harm. Beyond their responsibility for direct harm, dyscompetent professionals can have negative impacts on group performance. The optimal integration of professional accountability and quality improvement systems merits further investigation.

## Introduction

In 1989 Donald Berwick sounded a clarion call for continuous improvement in systems of care [1] and launched a movement that eventually found its foremost articulation in the Institute of Medicine’s *Crossing the Quality Chasm* [2]. Central to his argument was a tenacious critique of what he called the Theory of Bad Apples, whereby “quality is best achieved by discovering bad apples and removing them from the lot.” While laying the groundwork for our current emphasis on systems theory, Berwick also castigated the Bad Apple Theorists who “measure their success by counting heads on platters,” thus challenging the heart of that era’s professional accountability systems.

A brief debate ensued in which the Institute of Medicine reaffirmed the profession’s obligation to self-regulate, citing evidence that 5–10% of physicians are incompetent and/or otherwise problematic and asserting that “One can shift the curve by removing outliers from the professional community when their abnormal practices are truly extreme and constitute a significant percentage of the community’s practices” [3]. The profession’s leading quality champions, however, accepted Berwick’s critique and turned away from the discussion of problem physicians; they moved on instead to the enormous challenge of shifting healthcare’s mindset away from the illusion of physician responsibility for error-free patient care toward recognition and correction of system design flaws [4]. Professional self-regulation came to be seen as ethical but ineffectual, as summarized here by Troyen Brennan: “Although the ability to revoke the licenses of physicians who abuse drugs, have sex with their patients, or commit gross infractions is clearly helpful, I cannot cite any evidence that the general quality of medical care is improved by this method of removing a few bad apples” [5].

In retrospect, arguments for continuous improvement to the exclusion of outlier identification have created an unfortunate dichotomy between these approaches. Several physician leaders have more recently acknowledged that health systems can subscribe to both continuous improvement and rigorous professional self-regulation, developing workforce systems that address problem physicians [6] while balancing safety principles with accountability [7]. In 2013 Shojania and Dixon-Woods asserted that the profession has “probably reached the point where we can at least name the problem of bad apples without detracting from still crucial efforts to improve the design of organisational systems and human factors” [8]. In a 1990 paper Berwick himself hastened to validate the role of peer review professionals, analytic reviews, and disciplinary actions as needed, but this clarification had limited reach [9], whereas his 1989 commentary dichotomizing continuous improvement and bad apple theories has been by far the most-cited publication of his career. The force of Berwick’s original critique lingers, such that in 2015 Ezekiel Emanuel could still claim, without substantiation, that “one of the great understandings of the last few decades is the fallacy of the ‘bad apple’ theory of problems in medicine” [10].

In part the controversy has turned on empirical questions regarding the magnitude of physician dyscompetence, defined as a failure to maintain acceptable standards in one or more areas of professional practice [11], as well as questions regarding the efficacy of accountability processes. The impact of outlier removal, referred to in these debates as trimming the left tail of the performance distribution, remains unknown. Defenders of accountability are disadvantaged as well by the inherent gravity of decisions regarding a peer’s clinical privileges and potential exit from an organization or the profession, in contrast to the buoyant vision and optimism of quality improvement. Finally, there is little recognition of the overlaps and interplay between accountability processes and quality improvement, in particular the shared infrastructure of people, policies, and information systems that support both. Throughout our narrative we will employ the commonly understood distinction between accountability and quality improvement but then return to discuss their overlaps and interplay. We take accountability to encompass processes involving entry into the profession or organization, privileging, maintenance of license and certification, peer review, outlier identification, remediation, and discipline; self-regulation refers to such processes when primarily under physician control.

In the case study that follows, we present evidence that a focus on problem physicians through accountability and peer review systems led to a startling decrease in population mortality before additional continuous improvement measures took effect. We also suggest that the distribution of physician dyscompetence is skewed rather than bell-shaped. The case is extraordinary for being set in the California prison system and for involving a large number of problem physicians, but it raises issues that are critical to current discussions of professional accountability and self-regulation.

We begin with a brief description of the scandal that prompted unprecedented federal intervention [12], then present the mortality analysis. Next we describe the sequence of activities leading up to and following the decrease in mortality. The chronological sequence fortuitously isolated workforce accountability, including peer review and other professional self-regulation processes, from subsequent quality improvement initiatives and thus allows us to argue that the decrease in mortality was due to the systematic focus on problem physicians. We go on to point out that the commonly understood dichotomy between accountability and quality improvement belies their shared prerequisites and processes. It may now be possible to embrace both within a coherent pursuit of high performance and population health.

### Bereft of both quality improvement and professional accountability

In 1979 California housed 22,632 inmates in 12 prisons [13]; by 1999 the inmate population had grown to 160,687 and the number of prisons to 33 [14], the majority now in isolated desert locations. By many measures, the system was in shambles. A class action lawsuit filed in federal court in April 2001 argued that inmates were "not receiving constitutionally adequate medical care as required by the Eighth Amendment to the U.S. Constitution and that defendants are not complying with the Americans with Disabilities Act (ADA) and § 504 of the Rehabilitation Act" [15]. Following extended courtroom negotiations regarding medical care quality, the state and inmate plaintiffs agreed in 2002 to a remedial plan to be monitored by inspections and audits. That plan failed, however, and in 2005 the state acknowledged that “an inmate in one of California’s prisons dies needlessly every six to seven days due to constitutional deficiencies in the … medical delivery system” [16]. In consequence Thelton Henderson, the presiding federal judge, placed the prison medical system in receivership and ordered a reduction in the prison population [17], which from 2006 through 2008 exceeded 170,000 inmates.

In addition to glaring system dysfunctions such as medical records “either in a shambles or non-existent” and pharmacy “in almost complete disarray,” these deficiencies included “a prevailing lack of accountability” [16]. The state’s own expert witness conceded that “historically the CDCR [California Department of Corrections and Rehabilitation] would hire any doctor who had ‘a license, a pulse, and a pair of shoes’” [16]. Many states have a documented history of collaboration between medical boards and correctional systems to send physicians with restricted licenses into prison medical care [18, 19]. In California’s prison medical care system, the presence of physicians under medical board discipline was rampant [20]. While Judge Henderson acknowledged the presence of a cadre of highly qualified and dedicated clinicians, he concluded that even these clinicians were working ineffectively in a system that was “broken beyond repair.”

Under the terms of the receivership, the court recruited a new leadership team with a mandate to improve mortality and overall quality in the medical delivery system. To guide the transformation efforts, the leadership team relied on the Institute of Medicine’s *Crossing the Quality Chasm* report [2] and launched a series of interventions to address deficiencies in the structures and processes of care delivery, including human and physical resource deficiencies. Although the receivership continues, this bundle of accountability and quality improvement interventions has proven effective [21]. In the analysis that follows, we focus particularly on the years 2005 through 2007 and the impact of removing problem physicians.

## Methods

To investigate the effects of interventions before and after the receiver’s takeover, we analyzed quantitative data from public reports and internal databases and corroborated our findings with qualitative data from a rich trove of archival sources. Particularly useful were the receiver’s annual death review summaries with case descriptions including patient ages, diagnoses, and evidence of clinical mismanagement [22]. These and the other public documents are unusually revealing of processes normally kept confidential; they include hundreds of detailed case summaries, often with actual names of patients and physicians, as well as many other shorter vignettes.

We focused on mortality from 1998 through 2009 as an outcome measure because the California Department of Corrections and Rehabilitation (CDCR) had consistent and reliable internal and external death reporting processes throughout this period; reliable measures of morbidity were lacking. The CDCR cataloged each inmate death in an internal database as natural, accidental, suicide, homicide, or execution. Analysis for this study was done with de-identified data (S1 table). In our primary analysis we excluded suicide, homicide, and execution and merged accidental deaths into the natural category. The portion of inmates age 60 and older increased from 1.2% to 3.4% during this period, so we adjusted for age using age, sex, and total population data (S2 Table) from the CDCR’s public semi-annual census reports [23]. We set the 1998 age distribution as standard and used the direct standardization method to achieve comparability across subsequent years.

To test for shifts in age-adjusted mortality over the 1998–2009 period, we employed change point analysis using Taylor’s Change-Point Analyzer Software [24], which combines cumulative sum charts (CuSum) and bootstrap methods to detect statistically significant changes in time-series data and calculates a confidence interval for the dates of any changes. Change point analysis is being increasingly used in healthcare for time-series analysis [25, 26].

In an effort to make sense of these mortality results, we correlated them with multisource quantitative and qualitative evidence using a case study approach [27]. Where possible, we relied on the records of federal court proceedings and evidence submissions because of their credibility and availability. Although stakeholders disputed issues of control and strategy, there were few disputes over facts in this high-profile case. These documents thus serve as highly credible data sources, and most continue to be easily available via internet. Furthermore, they adequately reflect the multiplicity of voices relevant to most of our concerns here. They do vary, however, in the degree to which they adequately address all our concerns. For example, the annual death review summaries directly address the mortality results, and the receiver’s reports to the court offer a detailed history of clinical interventions, both of which are central to our study. These and other archival documents offer only indirect evidence regarding on-the-ground staff morale and professional norms, however, so here we must draw conclusions with more caution.

As we will describe in more detail below, the death review annual summaries were based upon the standardized death reviews performed by a team of the CDCR physician leaders. The physician reviewers included a determination as to whether the death was preventable and whether there were any simple or extreme departures from the standard of care. They also developed a taxonomy of lapses in care with 14 categories covering both individual lapses, e.g., failure to recognize “red flag” signs and symptoms, and system lapses, e.g., medication delivery error. Beginning with the year 2006 deaths, an outside expert hired by the receiver analyzed the individual death reviews and produced annual death review summaries, including clinical synopses of individual deaths deemed “preventable” or “possibly preventable” as well as year-over-year patterns of lapses of care and deaths.

In July 2006 the receiver began filing regular reports to the court, first on a bimonthly basis then decreasing in frequency to three times per year, in addition to strategic plans filed in 2007 and 2008. These voluminous reports and plans enabled us to establish a detailed sequence of policy decisions, initiatives, and improvements. Our analysis correlates this unusually reliable narrative construction of organizational change with the quantitative changes in mortality. We will describe additional archival materials in context below.

Finally, while our analysis rests upon strong, publically available sources, our archival research is informed by an ethnographic sensibility [28] facilitated by the experience of the first author (TEH), who had conducted research in the California prison system prior to leading the receiver’s clinical transformation initiatives. He chaired the administrative governance committee overseeing peer review but did not instigate the death review or peer review processes, nor did he participate in peer review deliberations. Notably, he came with a background steeped in patient safety and “Quality Chasm” philosophy and was a vocal critic of what he perceived as excessive reliance upon the death investigations and bad-apple pursuits that found favor in the courtroom setting. Only in retrospective reflection did he come to appreciate the contribution of accountability to the mortality reduction and the possibility of integrating these approaches.

This work meets the criteria for nonresearch public health activities exempt from ethics review as outlined by the U.S. Centers for Disease Control and Prevention [29].

## Results

**Fig 1:**
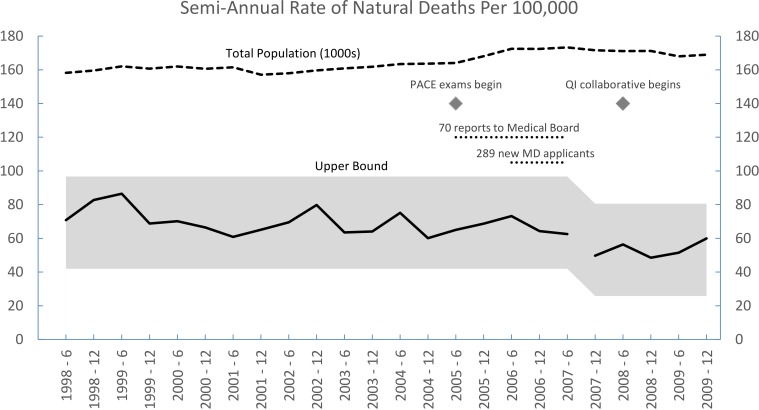
Change point analysis of age-adjusted natural deaths in California prisons. The mortality data series shows semi-annual age-adjusted natural death rates for years 1998–2009. The shaded areas reflect the range of expected rates at each time point. A single statistically significant change occurred in 2007. The semi-annual average rate of natural deaths decreased from 69.3/100,000 in the 1998–2006 period to 53.2 in the 2007–2009 period. The diamonds illustrate the time interval between effective accountability process, e.g., exams at the Physician Assessment and Clinical Education (PACE) program, and effective quality improvement (QI) programs. The dotted lines mark the time intervals for illustrative key events.

Table 1 shows the semi-annual population figures, total deaths, natural deaths, age-adjusted total death rates, and age-adjusted natural death rates from 1998 to 2009. The change point analysis in Fig 1 shows a plot of age-adjusted natural death rates and indicates that a single significant change in mortality occurred during this 12-year period, beginning in 2007. On an annual basis, mortality decreased from 138.7 per 100,000 in the 1998–2006 period to 106.4 per 100,000 in the 2007–2009 period. The confidence level for the significance of this change is 98%, and the 95% confidence interval for its timing is between December 2006 and December 2007. Repeat analysis including suicides and homicides (not shown) found a single change at the same point with a confidence level of 95%. Joinpoint analysis [30] yielded a best-fit slope that confirmed a single inflection in the mortality trend beginning in 2007.

**Table 1:** California prison mortality, 1998–2009.

				Semi-Annual Age-Adjusted Mortality	
Year	Population	Deaths	Natural Deaths	Deaths/100,000	Natural Deaths /100,000
1998 June	158,207	131	112	82.8	70.8
1998 December	159,563	151	136	92.0	82.7
1999 June	162,064	166	147	98.1	86.5
1999 December	160,687	139	122	79.4	68.8
2000 June	162,000	144	131	77.7	70.1
2000 December	160,626	136	125	73.5	66.5
2001 June	161,470	138	118	73.2	60.9
2001 December	157,096	150	127	80.2	65.1
2002 June	157,972	161	140	82.8	69.5
2002 December	159,654	176	166	85.5	79.8
2003 June	160,838	170	142	79.7	63.5
2003 December	161,798	162	140	77.7	64.1
2004 June	163,381	188	169	87.3	75.2
2004 December	163,634	160	142	71.5	60.1
2005 June	164,034	176	150	78.9	65.0
2005 December	168,055	201	173	84.2	68.7
2006 June	172,508	233	195	93.4	73.2
2006 December	172,379	196	172	78.5	64.3
2007 June	173,274	212	185	78.0	62.5
2007 December	171,568	185	155	66.5	49.7
2008 June	171,069	192	169	70.4	56.4
2008 December	171,161	177	155	59.1	48.5
2009 June	167,981	189	174	60.8	51.5
2009 December	168,905	207	189	69.4	60.0

The annual death review summaries allowed us to correlate changes in death rates, causes, preventability, and lapses of care with the sequence of efforts to implement reforms. As noted in the summaries themselves, there are multiple factors that limit quantitative year-to-year comparisons about preventability and lapses in care. Notwithstanding these limitations, the data on preventability and lapses in care, together with the individual case descriptions, yielded valuable insights.

As documented in the annual death review summaries, the causes of death in prison differed from national patterns as expected, with excess deaths from liver disease, drug overdose, suicide, and violence. Cancer was consistently the leading cause of death, exceeding cardiovascular disease. The major decrease by condition over time was in preventable cardiovascular mortality, which dropped steadily from over 10 per 100,000 in 2006 to 5 per 100,000 in 2009 [31]. Analysis by site identified only one outlier, consistent with the presence of a hospice program at that site.

Analysis by lapses in care included only those departures from the community standard of care that were deemed egregious. Lapses varied in dose-response fashion with judgments of preventability. In 2007–2009 the number of lapses in “preventable” deaths was 3.9; in “possibly preventable” deaths, 2.1; and in “non-preventable” deaths, 0.6 [32]. These dose-response correlations lend credibility to the reviewers’ assessments of both preventability and lapses. The 2008 annual summary noted, “These findings support the idea that adverse outcomes, in general, are a consequence of multiple errors: the Swiss cheese model of adverse events in which multiple ‘holes’ in the system all line up” [33]. The most common lapse in care, by far, was the failure to identify and assess “red flag” signs and symptoms. Improvements in this area may have been responsible for the measurable decrease in preventable cardiovascular deaths noted above. Cardiovascular conditions and asthma are among the conditions most quickly vulnerable to severe departures from the standard of care and thus most responsive to improvements in professional workforce performance. In contrast, lapses in care may have hastened death or increased suffering in patients with cancer or other terminal illness, but these deaths were classified as non-preventable.

The annual summaries also correlated the timing of reform initiatives with the progress of interventions. The summary written in 2008 linked improvements in mortality to changes in workforce. It was not until the summary of 2012 that the outside reviewer correlated decreases in lapses in care with the receiver’s multiple improvement interventions other than workforce. In the narrative that follows, we elaborate on the evidence for these correlations.

### The implementation of physician accountability

Inspection of Fig 1 suggests a modest improvement in mortality trend from 1998 to 2006 and a significant change in 2007. Court oversight, court-mandated improvements in mental health services, and isolated practice improvements [34] may have contributed to the initial modest decline. The federal court takeover and formation of the receivership team occurred in mid-2006. While it may be tempting to link the receivership’s quality improvement interventions with the significant mortality decrease in 2007, our narrative analysis of the years 2004 to 2007 reveals that the reasons for the downturn must have preceded those interventions. The decrease in mortality appears to be associated with implementation of accountability and peer review systems, i.e., with workforce, rather than the receiver’s quality improvement initiatives that came later.

Beginning in 2004, the court focused heavily on death reviews and the inadequate care they revealed. Court-appointed experts brought physician incompetence to light and described inadequate physician credentialing, supervision, peer review, and discipline. Many of the physicians had checkered or criminal pasts, and peer review was “bogus” or “not done at all” [16]. The state then contracted with the Physician Assessment and Clinical Education (PACE) program at the University of California, San Diego, to provide competency testing and remediation, if appropriate, for its primary care physicians. The state agreed to hire only board-certified or “board-eligible” physicians and began to send physicians without time-limited certification to the PACE program for testing. The department’s physician leaders initiated a broad-based peer review process that was centralized statewide.

The death review findings often brought physicians to the attention of the peer review committee, which supplemented that evidence with pattern-of-practice reviews of up to 60 charts, PACE assessments if available, and interviews with the physicians of interest if appropriate [35]. The committee could remove physician privileges if warranted by standard of care deviations and additional evidence. From June 2005 to July 2007, the committee took adverse actions against 56 physicians [35]. By law, peer review privileging actions, civil judgments, settlements, and criminal convictions require reports to the Medical Board of California. Table 2 shows that in fiscal years 2005–2007, the California Department of Corrections and Rehabilitation filed 70 reports, nearly two orders of magnitude more than the statewide average rate. Additional voluntary departures occurred once staff realized that increased scrutiny was inevitable and privileging actions a possibility. An internal memo described the exodus as follows:

“Of the first 53 physicians completing the PACE assessments by May 2006, 18 passed, 3 failed outright, and 32 passed with recommendations for remediation.… Of the 79 physicians who separated from state service from December 2004 to April 2006, only 10 were board certified and in good standing.”

The resulting vacancies were filled largely by physician contractors (not state employees) until the receiver raised salaries, included a differential for time-limited board certification, streamlined the hiring process, and intensified recruitment efforts. These measures led to an increasing number of physician applicants who successfully passed the initial hiring examination: 184 from March 2006 to March 2007 and another 105 in the next two months [36].

**Table 2:** Reports to the medical board of California.

	Fiscal Year 2005–2006		Fiscal Year 2006–2007	
	Reports filed	Reports per thousand physicians†	Reports filed	Reports per thousand physicians†
Reports from CDCR*	31	84.2	39	106.0
Total reports statewide	138	1.1	127	1.0

*CDCR, California Department of Corrections and Rehabilitation.

†During this period there were 368 physician positions approved in CDCR, although less than 300 were filled. There were approximately 130,000 physicians licensed by the Medical Board of California.

### Accountability processes preceded quality improvement initiatives

**Fig 2:**
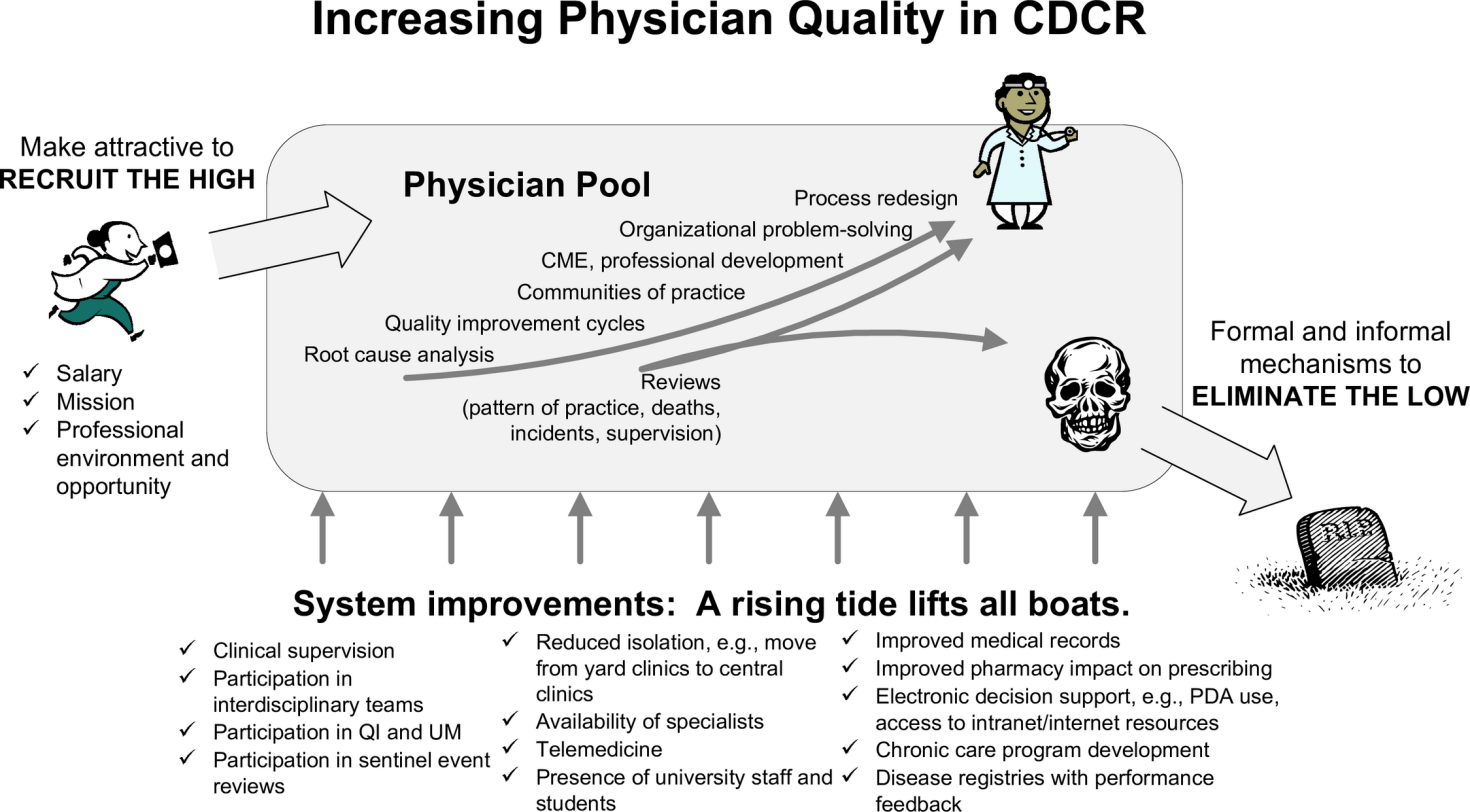
Driver diagram of interventions to improve physician performance in the California Department of Corrections and Rehabilitation (CDCR). Recruiters emphasized salary, mission, and a practice environment of renewed professionalism. Peer review enabled removal of truly problematic physicians. Although this 2006 diagram lists numerous system improvements designed to boost patient safety and professionalism, the only interventions that were mature in 2006 were the salary increases and processes for physician accountability. CME, continuing medical education; QI, quality improvement; UM, utilization management; PDA, personal digital assistant (mobile device).

The receiver also launched a host of initiatives to improve system performance, described at length in plans submitted to the court [37, 38] and illustrated in Fig 2. The receiver’s regular reports to the court have documented successful implementation of virtually all these quality and infrastructure initiatives [21]. What is now glaring, however, and came as a surprise to the first author, is that the decrease in mortality occurred long before these initiatives became relevant. In spite of the receiver’s broad powers and financial resources, program implementation was slow. Both the physical facilities and the bureaucracy could be almost unimaginably inhospitable and intransigent. Connecting stand-alone computers in all the clinics took three years, delaying deployment of an electronic pharmacy system. A small physician leadership team attended its first quality improvement training from the Institute for Healthcare Improvement in July 2007. The first interdisciplinary chronic care collaborative, focused on asthma, began in June 2008 with 6 prisons; the other 27 prisons were engaged in 2009. The newly created quality, utilization management, and public health units installed their first physician directors in 2008. An executive in charge of reforms in laboratory, radiology, and ancillary services began work in late 2009. Accreditation for continuing medical education came in 2009. In summary, implementation of the receiver’s “Quality Chasm” portfolio began in earnest in 2008–2009. Development of statewide systems for professional accountability and peer review, in contrast, began in 2005 and accelerated in 2006–2007. Physician competency testing at the PACE program began in March 2005, and remedial training began in June 2006. These accountability processes and workforce improvements preceded the drop in mortality seen in Fig 1.

The annual summaries of death reviews provide corroborating evidence of excess mortality linked to physician performance in 2006 followed by improvement in 2007. In 2006, 18 deaths were deemed “preventable” and another 48 considered “possibly preventable” [35]. Physicians made an array of errors leading to 6 asthma deaths, for example, and failed to respond to classic cardiac signs and symptoms in another 6 deaths. One inmate died of a perforated ulcer after presenting multiple times over 5 days; another died of acute pancreatitis after presenting 9 times over 3 days; and another died of incarcerated hernia after physicians delayed 5 weeks before referral to a specialist in spite of recurrent abdominal pain, vomiting, and known inguinal hernias. In 2007 the physician leadership refined the process for determining whether deaths were preventable; they also developed a taxonomy for identifying lapses in care at both the individual and system level, as described above. The most common serious lapse was the “failure to recognize or evaluate important signs and symptoms,” consistent with the importance of diagnostic errors as described by patient safety advocates [39]. Reflecting death data through 2007 and peer review data through July 2008, the annual summary published in November 2008 concluded:

“The major clinical impact felt throughout the system … has been from improvements in the number and caliber of healthcare professionals. … Most of the Receiver’s other interventions that aim to improve the quality and cost-effectiveness of healthcare services are still in early, pilot, or planning stages. … Death rates in the CDCR are significantly decreasing in part because the high-quality … peer review process has resulted in the replacement of 85 potentially dangerous providers with new well-qualified providers” [33].

As described above, the receiver’s quality initiatives took shape in 2008 and gained traction gradually over the next several years. In 2012 the outside reviewer could finally point to these as responsible for an overall decline in serious lapses in care [40].

### Recovering professionalism

In addition to the direct harms inflicted by problem physicians, the court’s uncontested 2005 findings document pointed to harms induced by “a culture of non-accountability and non-professionalism.” The court’s message to qualified and dedicated physicians “who have been struggling to provide quality care in dire circumstances” was that “California is about to embark on a dramatic transformation of its prison medical system” [16]. We have correlated the mortality decrease with implementation of workforce accountability processes and replacement of problem physicians by qualified physicians, but it would be helpful to know more about changes in social dynamics and norms in the years 2005 through 2007. The archival documents offer little direct and systematic evidence regarding staff morale and professional norms, but there is indirect evidence of transformation.

The baseline was grim. Court experts in 2006 cited the frustration of managers with their inability to discipline staff and the ploys used by poor performers, which were “demoralizing to both managers and staff who are working hard” [41]. Plaintiff attorneys routinely inspected prisons and wrote detailed, structured reports about medical facilities, staffing, and processes. A review of 31 such reports revealed significant changes in local medical staffs from 2006 to 2007. In 2006 the reports commonly used language such as “still in crisis” and “mired in the same problems.” In one prison, some physicians were routinely working less than a full day, but “management seemed at a loss,” afraid that “if individuals were disciplined, they would not be able to fill the positions at all.” Only two of the 13 reports from 2006 used positive descriptors, e.g., in October 2006 managers and medical staff in one prison were “excited about the improvements that have occurred.” In contrast, 13 of 18 reports in 2007 used positive language about diverse prison medical staffs such as “strong commitment,” “steady improvement,” “proud of the achievements they had made, and with good reason.” At another prison, “the hiring landscape has dramatically changed for the better, with contract physicians now applying for permanent state jobs.”

Similarly, the receiver’s June 2007 report about San Quentin cited the improved stability of the medical staff, the recruitment of recent graduates from the University of California, San Francisco, and the new interest from contract physicians in becoming state employees [36]. A declaration submitted by the first author in July 2007 with regard to other prisons asserted, “Bit by bit, the local prison staff are becoming enthusiastic about the realistic possibilities for significant change” [42]. In a 2008 declaration he could more confidently assert, “there has been a change in the attitude of healthcare staff, many of whom are voicing newfound empowerment and recommitment to their original motivations as healthcare professionals” [43].

These various reports suggest aggregate improvement in morale and restoration of professional norms; they also allude to the potential downsides of accountability implementation. As noted in the introduction, peer review in stable systems has been characterized at times as a heads-on-platter exercise. In this unstable system there was widespread concern that the court’s focus on weeding out problem physicians, particularly in the context of death investigations, would lead to unfair blame of individuals, sometimes referred to as “collateral damage.” The following description from the receiver’s September 2007 report [44] is terse but nevertheless conveys the gravity of these processes:

The committee met 14 times from June 2007 to September 2007 and reviewed 33 initial allegations of clinical misconduct or neglect and the findings of 23 peer review investigations. The committee acted by suspending the privileges of 12 practitioners and restricting the privileges of an additional three physicians. They also restored the privileges of two physicians.

In 2006 and 2007, the receiver’s team revised the death review process to incorporate safety principles and developed a taxonomy of lapses in care to assist with identification of system issues contributing to adverse outcomes [33]. In order to promote fairness and allay rising staff anxiety, the receiver’s team introduced “just culture” principles and promoted use of James Reason’s decision tree for determining culpability of unsafe acts [45]. The team also hastened to deploy professional development programs for all physicians, as illustrated in Fig 2, in addition to remediation opportunities for marginal physicians. Given that good physicians were often “isolated among those considerably less competent and/or less dedicated than themselves” in a system with “few support and safety mechanisms in place,” these changes helped mitigate medical staff concerns that heightened accountability processes could indiscriminately sweep up marginal or even fully competent physicians [37].

The physician union repeatedly expressed concern about physicians being unfairly blamed for bad outcomes. There were also complaints of discrimination against minority physicians; given evidence of continued discrimination and associated career dissatisfaction in medicine at large [46], this constituted another major concern. It is noteworthy, however, that the union repeatedly expressed support for efforts by the court and receiver to restore professionalism and in particular for a thoughtful peer review process independent of monitoring and control by non-physician state employees [36]. In spite of the evident anxiety and consternation caused by implementation of accountability processes and the potential negative effects of this anxiety and consternation upon staff morale and relationships, it appears that norms improved, and the fact remains that mortality decreased.

## Discussion

In this case study, implementation of robust processes for professional accountability was associated with a significant decrease in population mortality well before other system improvements were in place. In the discussion that follows, we explore the conceptual implications of this evidence in light of the historical debate outlined in our introduction. We do not claim that similar reductions in mortality would obtain in another delivery system or the medical profession at large, but we do believe that this experience can help reinvigorate practical and scholarly attention to accountability and professional self-regulation at the organizational level. Following discussion of the organizational implications, we hazard several observations regarding the profession at large.

The impact documented in this case study challenges the assumption that professional self-regulation is ineffectual. As ardent supporters of systems thinking, we by no means devalue comprehensive quality improvement initiatives, which were eventually effective in this setting. Rather, our analysis argues against a dichotomous opposition between professional self-regulation and continuous improvement. The perception of dichotomous opposition is deeply ingrained; Berwick’s initial argument has settled into orthodoxy, unmoderated by his later clarification, in spite of the safety movement’s familiarity with Reason’s decision tree for determining culpability [45]. Champions of quality and safety have been slow to acknowledge that workforce accountability and quality improvement can be synergistic. To be successful, both require systemic implementation of enabling expertise, policies, processes, and staff resources. In retrospect, conceptual rapprochement should have been easier in this setting than it was. Fig 2 suggests overlaps and synergies, and the 2007 strategic plan builds on the Baldrige framework, which seamlessly integrates a focus on workforce into a model of organizational change. Berwick himself had championed the Baldrige framework in his 1990 paper that argued for a partnership between peer review and quality improvement.

### Professional self-regulation within organizations

Given the long-standing, oft-cited problems with peer review [47], its achievements in this system were noteworthy. The peer review committee reviewed patterns of practice, not just single incidents, and solicited input from multiple sources, including the physician under review. With input and eventual support from the physician union, the governing body developed a due process procedure that withstood legal challenge [48]. As within most organizations, many of the same physicians led both the peer review and quality improvement efforts.

A recent primer for creating a “thoughtful, fair, systematic, and organized approach” to peer review [49] noted the surprising lack of literature on the topic. We share the concern about scholarly neglect of this domain. Most discussion of peer review and professional self-regulation fails to account for the evolving organizational forms of our current delivery systems, as if hospitals and medical boards were the only loci of activity. Health plans and many medical groups, clinics, and ambulatory surgery centers perform formal peer review, as does the occasional post-acute or long-term care provider, all overlaid with a now-bewildering array of contractually-accountable entities that also review individual physician performance. Peer review systems can operate across an entire state, as in this case study, or even across the nation [50].

Macro-level policy discussion about professional self-regulation, e.g., maintenance of certification, reveals antagonistic divisions between the profession’s guardians of competency and the rank-and-file [51]. At the micro-level within organizations and delivery systems, however, the available evidence suggests that these contentious debates do not necessarily reflect physicians’ everyday attitudes and behaviors. One survey found that physicians are “disheartened” when their efforts to discipline fellow physicians are thwarted [52]. Another study has described how perceived violations of clinical standards across specialties within a hospital can trigger moral emotions that mobilize individual and/or collective action, including a range of corrections and “other-condemning” penalties directed toward the offending physicians [53]. This qualitative study found that rank-and-file physician behavior was explicitly motivated by concern for patients and respect for professional values. The study did not document physicians’ reactions to overt dyscompetence, which presumably would trigger yet stronger emotions and actions.

**Fig 3:**
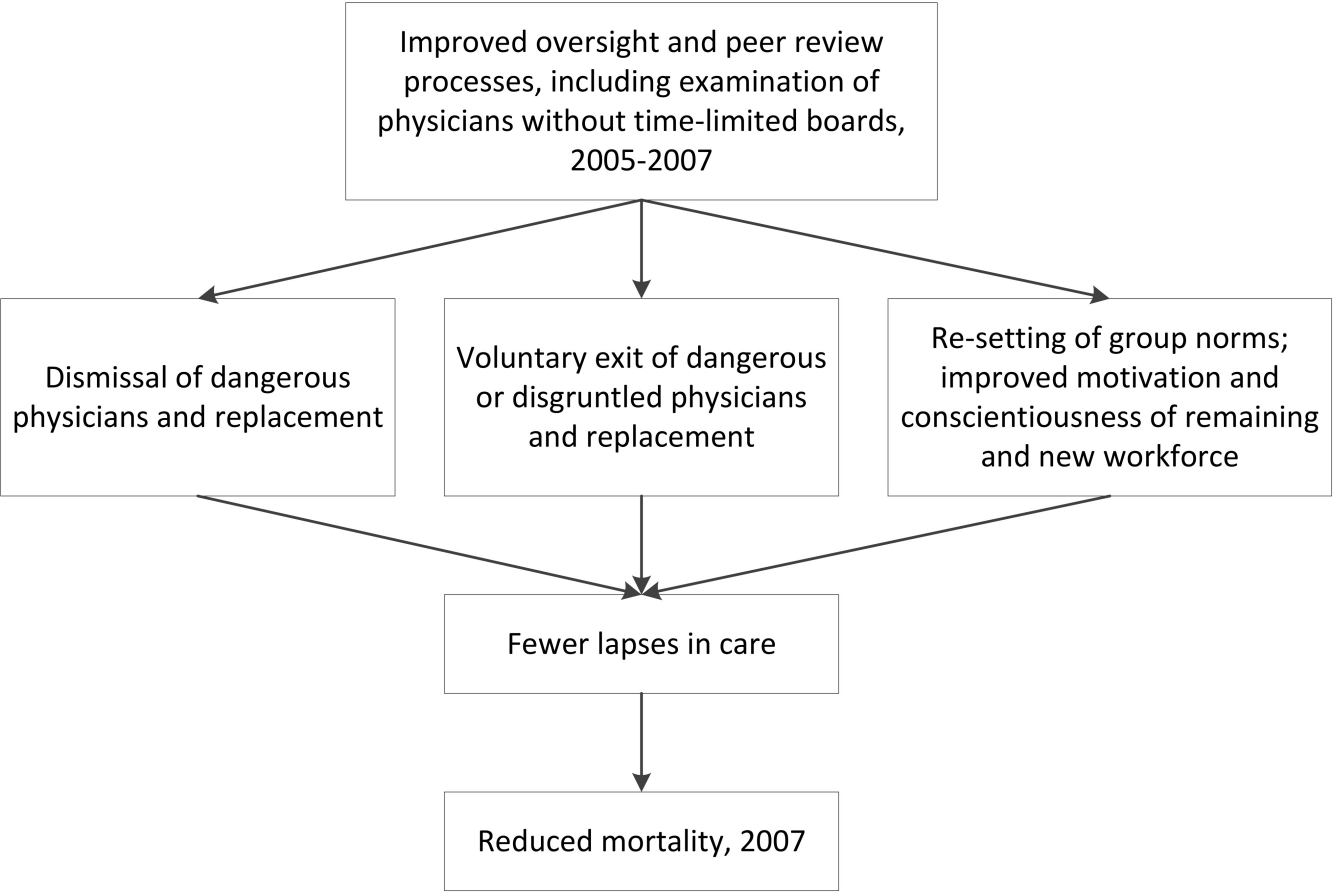
Drivers of mortality reduction.

The comprehensive and methodical nature of the peer review processes deployed in our setting may have mitigated against the perception of arbitrary injustice and assisted in winning physician support. While we have alluded to anxieties induced by peer review, the physician leaders and union all came to support it. We also found indirect evidence of improvement in professional norms. The perception of procedural justice has been associated with improved clinical performance [54], and evidence from social science suggests that use of appropriate sanctions with marginal and/or difficult employees can elevate group norms and improve motivation [55]. As shown in Fig 3, this dynamic may have contributed to the reduction in population mortality. Beyond the direct harms done by dyscompetent professionals, these individuals can have significant negative impacts on group performance, e.g., lowering group morale, trust, resilience and creativity, thus contributing to burnout. Individuals who are skilled but unconscientious in their work have disproportionately negative effects compared to individuals who are well-meaning but lacking in skills [56].

The reluctance of organizations to discipline physicians, even in “egregious” cases [50], is widely known, as is their reluctance to report physicians who are disciplined. A 2017 study of five Veterans Administration Medical Centers found that only one of nine disciplined physicians were reported to appropriate boards. Most of the physicians not appropriately reported were “likely” practicing elsewhere; one of these subsequently had privileges revoked by another hospital for the same cause [57]. A study commissioned by the California legislature found that organizations often pressure physicians to resign in ways that do not trigger formal reporting to the medical board: “The costs of [reporting] are prohibitive, and entities and physicians use all possible means to avoid the time and money that are involved in the lengthy, contentious processes” [52]. This study found that non-hospital entities were even more reluctant to investigate dyscompetence and pursue discipline: “Health plans and medical groups are generally passive and depend upon hospitals and medical boards to bear the heavy burden of pursuing physician discipline.” The steep costs of physician discipline, however, must be weighed against an organization’s direct and indirect costs of physician dyscompetence. An academic medical center reported that in a 5-year period a single neurosurgeon was responsible for 12 of the 23 lawsuits in which its neurosurgeons were named. This neurosurgeon “departed from the [department] during the study period” [58].

The experience of peer review here also challenges Berwick’s sharp 1989 distinction between “the truly avaricious and the dangerously incompetent” from “the rest of us.” The threshold of adequate performance is not clear-cut. Setting a threshold for competent performance is a very complex task, even when limited to narrowly defined clinical competence [59]. Remediation for physicians above the threshold for formal reporting is often possible and appropriate at the organizational level without triggering costly and cumbersome high-stakes due-process procedures. Some medical centers have recently developed novel processes to address performance issues that are clustered above the thresholds appropriate for peer review [60, 61].

Furthermore, the distribution of performance outside of standardized testing settings is not likely to be a normal, bell-shaped curve, as assumed by both critics and proponents of peer review. Such curves are a product of standardized test design [62]. Stephen Jay Gould’s examination of performance in baseball helps illuminate the challenge in medicine. As baseball improved in the twentieth century, variation among the better batters decreased, clustering against a “right wall” of high performance such that the right tail was truncated [63]. The left skew of poor performance, on the other hand, was relegated to the minor leagues. Most measures of real-life performance in Olympic sports [64], boating [65], and medicine [66, 67] also yield competence curves in which good and very good performance clusters against a right wall. Simply put, “the distance between the average and peak performance narrows over time” [68]. Medicine’s unresolved problem is the long left skew of dyscompetent performance. The left tail’s heterogeneity was evident in the system described here; it has also been reported from the PACE experience [69]. A study of physicians disciplined by the Medical Board of California described those physicians as “a subset of a poorly defined universe of physicians with significant practice deficiencies” [70].

### Self-regulation within the profession

The data in this case study address Brennan’s observation about the lack of evidence linking the removal of “bad apples” with improvement in overall quality of care. Similar improvements in overall mortality might obtain only in settings with high concentrations of physician dyscompetence and high-risk patients dependent upon those physicians. Although our results are not generalizable to the population at large, they do raise questions about the harms of dyscompetence. The exact magnitudes of physician dyscompetence and consequent patient harms are understudied and uncertain. The 1990 Institute of Medicine report cited earlier estimated that at any point in time, 5–10% of U.S. physicians are incompetent, impaired, or otherwise routinely perform below the standard of care [3]. More recent reviews concur [6, 11], suggesting that licensure alone is inadequate to assure acceptable levels of competence and performance. Canadian provinces, the United Kingdom, and Australia have profession-led or governmental systems for screening the performance of all physicians, screening a random sample, and/or targeting certain higher-risk categories, e.g., physicians over a certain age [71]. Physician accountability in the U.S. is non-systematic, dependent largely upon malpractice and the market [72]. On its face, maintenance of certification has limited impact since 20% of U.S. physicians are not certified by a specialty board [73].

The great bulk of problem physicians congregate where oversight is weak or non-existent, such as solo practice [74]. Hospital medical staff committees no longer have line of sight into the performance of most ambulatory care physicians. Oversight in nursing facilities and other long-term care settings has always been rudimentary [75]. These latter settings serve particularly complex patients who are vulnerable to standard of care violations. Variation in physician attentiveness and quality in long-term care settings is well-known [76]; the patient consequences of dyscompetence are unstudied.

Of note, the number of staff terminations in our study far exceeded the subsequent number of licenses lost, so as with the 2017 Veterans Administration study, it is likely that the majority of the problem physicians who left California’s prison system are now practicing elsewhere, with or without adequate oversight. The dilution of these dyscompetent physicians into larger state and national systems diminishes the measurable aggregate impact of their lapses in care, but it does not render negligible the harms they may inflict on patients. The peer review committee in this setting took action against physicians when the committee members concluded that these physicians were likely to continue committing egregious lapses in care and patient harm. Stakeholders may differ as to who is responsible for preventing such harm, whether from these physicians in particular or the broader 5–10% cited above. In its 2005 findings regarding physician incompetence in California prisons, the court identified “a single root cause of this crisis: an historical lack of leadership, planning, and vision by the State’s highest officials” [16]. On the other hand, the public firmly believes that the medical profession bears responsibility for disciplining itself [77].

Organizations such as hospitals and large medical groups can manage physician entry criteria and oversight, including remedial training and discipline as needed. Although most may still be reluctant to bear the cost and controversy of formal expulsion, some have fully integrated these processes with their quality improvement efforts. Respectful and conscientious peer review with ample procedural safeguards should no longer be denigrated as fallacious bad-apple thinking. Our broader challenge in the U.S. context is managing professional self-regulation outside the boundaries and scrutiny of these organizations.

### Limitations

We acknowledge that generalizations from this experience merit caution. Our report carries all the limitations of a retrospective case study, marked by an atypical setting, the extraordinary nature of the scandal and the subsequent institutional response, and a high concentration of problem physicians. We have not detailed the CDCR’s specific accountability processes, but good practical accounts of mortality review [78] and peer review [49, 79] are found elsewhere. Problems with the reliability of peer review are well-known [80], but our experience adds credibility to reports that structured peer review can distinguish good quality from bad [81, 82]. We have not elaborated on the workforce turnover among nurses or on the nursing-specific interventions. The sequence of accountability and quality improvement interventions in nursing paralleled those in medicine, but nurses were rarely responsible for lapses leading to deaths. We were not able to investigate the relationship of physician board certification to performance, nor did we have data on physicians’ beliefs and experience, which would enrich our explanations. Professional identity, including one’s commitment to the professional values and professional self-regulation, is multidimensional, varies widely across the profession, and interacts in complex ways with one’s work group and organization [83]. Physician leaders and staff in other settings may respond quite differently when faced with crises of professional legitimacy; their accountability systems, moreover, are likely to be far less robust. In addition to peer review with an enabling governance structure and robust legal support, initiatives here included thorough investigations of all deaths, disciplined root cause analysis, generous use of the PACE program, and investments in physician leadership training. Finally, we did not have data to age-adjust mortality rates beyond 2009 and thus evaluate the impact of then-emerging quality improvement initiatives; although reports cited above suggest they were effective, their impact in the subsequent time frame is beyond the scope of this study.

## Conclusion

These limitations notwithstanding, the core contribution of our study is its empirical evidence for the value of workforce accountability processes in preventing harm to patients. The finding of a significant decrease in population mortality is bolstered by evidence from the annual analyses of death reviews, which graphically illustrate the harm directly attributable to dyscompetent physicians. Our analysis draws attention to conceptual lacunae regarding safety and quality management; these lacunae may have potentiated the scholarly neglect of self-regulation in medicine. We agree with Shojania and Dixon-Woods that a vigorous research agenda should now move beyond the profession’s self-imposed taboo against discussing and managing the poor performance of individual physicians [8]. A data-driven discussion on professional self-regulation is needed and may now be practicable. As electronic record systems become more comprehensive and robust, creative research designs may be able to tease out the impact of various professional accountability systems and their optimal integration with other approaches to improving health systems performance.

## Supporting information

S1 Table Death ages, dates, and categories. (XLSX)

S2 Table Semi-annual age data of inmate population. (XLSX)
